# Thematic morning report in clinical training for critical care medicine resident physicians: Preliminary exploration in a tertiary hospital of China

**DOI:** 10.1371/journal.pone.0324871

**Published:** 2025-08-14

**Authors:** Lei Zhang, Na Li, Yu Liu, Yan Li, Zhengwen Liu

**Affiliations:** 1 Department of Critical Care Medicine, First Affiliated Hospital of Xi’an Jiaotong University, Xi’an, Shaanxi, China; 2 Department of Rehabilitation Medicine, First Affiliated Hospital of Xi’an Jiaotong University, Xi’an, Shaanxi, China; 3 Department of Teaching Administration, First Affiliated Hospital of Xi’an Jiaotong University, Xi’an, Shaanxi, China; 4 Department of Infectious Diseases, First Affiliated Hospital of Xi’an Jiaotong University, Xi’an, Shaanxi, China; University of Limpopo, SOUTH AFRICA

## Abstract

Morning report (MR) is one of the most important forms of resident standardized training. However, at present, it has not been carried out in most residential training bases in China. The specific content and implementation plan of MR lack standardized process. We introduced thematic MR in the training of residents who underwent standardized training in critical care medicine. A total of 37 residents (major in critical care medicine) were divided into two groups: the MR group (n = 22) and the traditional training group (n = 15). After receiving a 24-week training in the ICU of the Department of Critical Care Medicine, a comprehensive ability of critical care medicine was evaluated in the residents. The performance was assessed by Objective Structured Examination (OSCE) stations and theory examinations, including clinical decision-making ability, emergency treatment ability, clinical practice skills, communication and cooperation skills and medical theoretical knowledge. The results showed that, although there was no significant difference between the two groups in terms of communication and teamwork skills (*P* = 0.524), the MR group outperformed the traditional training group in terms of clinical decision-making ability, emergency treatment ability, clinical practice skills, medical theoretical knowledge (*P* < 0.05). Additionally, a questionnaire survey on the training mode of thematic MR in critical care medicine was conducted. The results showed that up to 90.9% (20/22) of residential physicians believed that thematic MR is necessary in the training of critical care medicine, and 86.4% (19/22) of them believed that the implementation of thematic MR could improve their mastery of knowledge in critical care medicine. In conclusion, this preliminary exploration demonstrated that thematic MR has the potential to enhance the training effect of residential physicians in critical care medicine. It is a favorite way among residents owing to its ease of implementation, flexibility, and hierarchical structure in the training of critical care medicine, offering a valuable complement to conventional training.

## Introduction

The importance of global competence has been acknowledged in medical care as well as medical education. Medical schools should conduct further educational reforms to promote students’ global competence [1].How to improve the competence of medical students ? Standardized resident physician training is an important part of post-graduation education for medical students. As it focuses on specific cases and patients, the main purpose of residency training is to train the thinking patterns of resident physicians through clinical practice, thereby enhancing their analytical and judgment abilities [2–3]. Clinical training should be based on evidence-based medicine, and its specific implementation relies on various forms of educational activities [3]. Taking internal medicine training as an example, in addition to self-education through observing and emulating patient management during postgraduate training, there are two fundamental and necessary forms of training, namely morning report (MR) and teaching rounds led by faculty [4].

MR is a daily educational activity that is part of the training for resident physicians in the United States [5]. Indeed, MR is a method used to train resident physicians, particularly those specializing in internal medicine, clinical reasoning and self-directed learning. It primarily involves daily discussions of interesting or challenging cases encountered during patient care, aiming to enhance residents’ abilities in disease diagnosis and management. The interactive nature of MR fosters critical thinking skills and encourages residents to actively participate in the decision-making process [6]. Through these discussions, residents can learn from the experiences and perspectives of their colleagues and attending physicians, ultimately improving their clinical skills and knowledge. MR have become an important way of improving specialist training [7], especially as case-based discussions supplement lecture-based educational activities [8] Studies have shown that MR improve both clinical reasoning and learning if all physicians participate in the discussions [9].

Heppe et al.[10] found that teaching during MR was typically prepared, moderated and presented by a junior doctor using a digital presentation, and that it tended to comprise a single case and include discussions about possible diagnoses. Sadie et al. consider that [11] MR is the predominant term for a case-based conference in which a chief resident (CR), senior resident, or faculty member guides residents and students through a discussion about a patient case or cases.Typically, MR is conducted as a small meeting lasting approximately one hour, held at regular intervals, often on a daily basis [12]. It is primarily attended by resident physicians with at least two years of training, and is moderated by the chief resident or attending physician. Cases for discussion are typically provided by the resident physician who is on duty in the preceding one or two days. The selection of cases focuses on common and frequently encountered diseases that are essential for residents to master according to the training requirements [11]. The structure of MR can be divided into three parts: (1) Prelude: A brief review of the patient’s medical history. (2) Detailed case discussion: This typically involves patients seen by internal medicine, pediatrics, and surgical resident physicians. Topics may include diagnosis, differential diagnosis, and discussions of diagnostic test results. (3) Conclusion: This may include feedback and analysis during the “teaching moments,” as well as identifying questions that require further research. By innovating the format of MR, it stimulates the resident physicians’ ability to engage in self-directed learning. It guides learners in interactive group discussions following a “search mode,” following the principles of learner-centered and reflective learning [13].

Currently, standardized MR has not been widely implemented in China’s standardized training bases for resident physicians. Therefore, it is necessary to explore the aspects of professional scope, implementation methods, timing, case selection, and standardized organizational format to achieve effective teaching outcomes. Critical care medicine trainees and faculty must acquire and maintain the skills necessary to provide state-of-the art clinical care to critically ill patients, to improve patient outcomes, optimize intensive care unit utilization, and continue to advance the theory and practice of critical care medicine [14]. The knowledge that resident physicians acquires can often become disconnected from clinical reality, making it difficult for them to properly understand and address specific issues. This results in a gap between theoretical knowledge and practical application. Standardized MR may not effectively address the complexity of patient conditions, the diversity of diagnostic and treatment modalities, and the extensive and fragmented knowledge covered by the cases. It fails to provide a focused and in-depth understanding and practical application of specific professional knowledge and skills, making the learning objectives less clear and unable to achieve the standardized and homogeneous training goals of standardized training for resident physicians in critical care medicine.

Therefore, we proposed a thematic MR training model that combines actual clinical cases in the workplace and focuses on specific knowledge points or professional skills. For example, the clinical application of mechanical ventilation can be discussed in a specialized manner. The discussions can start from the basics and gradually progress in depth, enabling resident physicians to improve their understanding of respiratory pathophysiology, respiratory function monitoring, and the use of ventilators, enhancing their professional skills step by step. Here, we report the preliminary exploration of thematic MR in critical care medicine resident training in a tertiary hospital of China.The objective of this study is to assess whether a structured Thematic Morning Report model improves critical care competencies in comparison to traditional training methods, emphasizing clinical decision-making, emergency response, and theoretical knowledge.

## Methods

We hypothesized that residents participating in Thematic MR would demonstrate superior performance in clinical decision-making ability, emergency treatment ability, clinical practice skills and theoretical knowledge compared to those receiving traditional training.

### Ethics statement

This study was approved by the Ethics Committee of the First Affiliated Hospital of Xi’an Jiaotong University (No. 2016−159). Written informed consent was obtained from all the participants of the study.

### Settings

This study was performed in the Intensive Care Unit (ICU) of the First Affiliated Hospital of Xi’an Jiaotong University. This hospital is a tertiary hospital in northwest China. It has a complete set of disciplines and accommodates 3,765 ward beds. The hospital undertakes multi-level and multi-major teaching tasks for medical education, including post-graduation medical education, continuing medical education and medical education of international students from 34 countries. The hospital is one of the first to carry out standardized training for resident physicians and specialist physicians. It is approved as “National Clinical Teaching Training Demonstration Center”. The ICU of the hospital has 72 ward beds and is one of the earliest established critical care medicine disciplines in the northwest region of China. The department provides critical care, resuscitation, life support, and organ function maintenance and support for critically ill patients from various clinical specialties. In 2018, it was approved as a standardized training base for specialized physicians in critical care medicine.

### Participants

To investigate the effect of thematic MR in critical care medicine training, we recruited 43 residents specializing in critical care medicine for standardized training between October 2020 and October 2022. These residents were randomly assigned in a 1:1 ratio to either the thematic MR group (n = 22) or the traditional training group (n = 21). However, due to the lengthy training period, 5 residents from the traditional training group choose to withdraw from the study. Ultimately, a total of 37 residents participated in the study, with 22 residents in the thematic MR group and 15 residents in the traditional training group. The thematic MR group implemented a training mode that incorporated traditional methods. Following a 24-week training in the ICU, all participants underwent an evaluation to assess their comprehensive abilities in critical care medicine.

### Concept of the thematic morning report

Thematic MR refers to the focus on a specific topic during the MR session. It aims to provide a comprehensive analysis of a particular category of clinical problems encountered by resident physicians. By highlighting key points and setting clear learning objectives, this approach enhances learners’ interest and engagement, while also training their clinical reasoning skills. Ultimately, it helps improve their clinical skills and learning confidence.

### The topics covered in thematic morning report sessions

Based on the recent clinical teaching experience of resident physicians in the intensive care specialty at our ICU training base, and considering the characteristics of critical care medicine, we have identified common problems, confusing knowledge points, and challenging areas that resident physicians often encounter in their clinical work. We have also identified the essential skills that they need to master. Table 1 presents the topics covered in our current stage of thematic MR sessions at our training base.

**Table 1:** The topics covered in thematic morning report sessions.

Knowledge module	Theme
Respiratory therapy	Oxygen therapy
	Blood gas analysis
	Non-invasive mechanical ventilation
	Basic mode and parameter setting of invasive mechanical ventilation
	Advanced mode and parameter setting of invasive mechanical ventilation
	Respiratory mechanics
	Respiratory rehabilitation
	Weaning of mechanical ventilation
Circulatory system	Non-invasive hemodynamic monitoring
	Invasive hemodynamic monitoring
	Vasoactive drug application
	Diagnosis and treatment of septic shock
	Fluid responsiveness and fluid resuscitation
Electrolyte and acid base disorders	Hyponatremia and hypernatremia
	Hyperkalemia and hypokalemia
	Hypomagnesemia
	Hypophosphatemia
	Metabolic acidosis
	Metabolic alkalosis
	Respiratory acidosis
Renal replacement therapy	Calculation of the therapeutic dose
	Calculation of the replacement fluid formulation
	Anticoagulation mode
	Monitoring and adjustment of citrate anticoagulation
Non-technical skills	Principles and contents of SBAR^*^ shift handover
	Tell bad news
	Communication ability cultivation

* SBAR is a structured method for communicating critical information that requires immediate attention and action. It has 4 steps: Situation, Background, Assessment and Recommendation.

### The implementation process of the thematic morning report

#### The form of the thematic morning report.

Time: between 9:00 am and 12:00 am from Monday to Friday

Duration: 30 minutes – 45 minutes.

Participants: Attending physicians, chief residents, and residents specializing in critical care medicine who were assigned to the thematic MR group who underwent standardized training in critical care medicine. The subgroup number of participants ranged from 6 to 10.

#### Determination of the theme.

Combined with the training standards and the clinical problems encountered in the work, the phased and progressive learning objectives are designed based on the degree of difficulty according to a training purpose, which is the theme of this round of MR.

#### Implementation process of thematic morning report.

**Fig 1:**
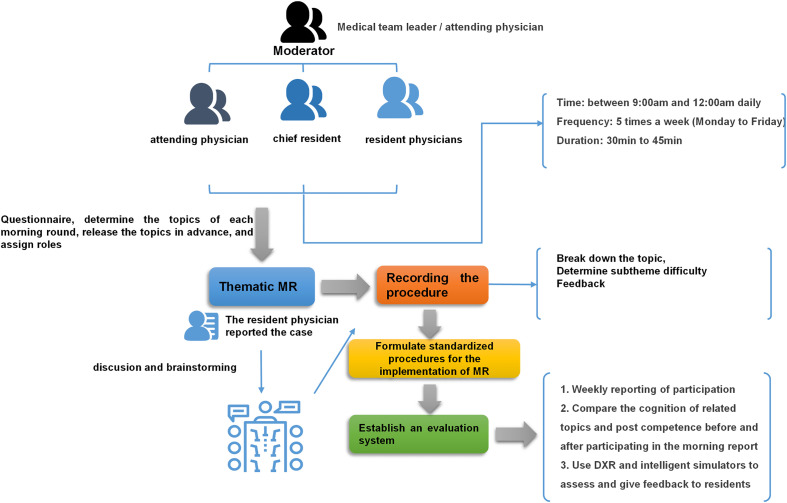
The specific implementation process of morning report.

(1) Determine the level of participants and the corresponding topic; assign roles to all resident physicians participating in the discussion (moderator, case reporter, recorder, order of participating in the discussion); and determine the medical team leader/ attending physician participating in the discussion.
(2) Conduct the theme MR, and record the whole process of the morning MR by the designated clerk: Step 1: The MR session starts with the facilitation of the attending physician or the leader of the medical team who announces the topic for the MR. They guide the resident physicians in presenting the basic details of cases related to the chosen theme that they have encountered in recent days. The resident physicians report the clinical issues they faced, the caring and treatment measures they implemented at the time, and the corresponding outcomes. They also have the opportunity to express any confusion or questions they have. Step 2: All the resident physicians involved in the discussion put forward their own analysis and opinions, and discuss with the relevant basic knowledge (they can search the Internet on site). Step 3: The attending physician or the leader of the medical team provides teaching feedback during which they highlight the relevant professional knowledge points and clinical reasoning key points related to the chosen theme. They also set goals for further learning and assign them to all learners. They designate the next moderator and topic for the following MR, following the pre-designed progressive learning content to increase the level of challenge. The specific implementation process is shown in Fig 1.

### Evaluation

Following standardized training in critical care medicine, participants underwent evaluation through Objective Structured Examination (OSCE) stations to assess practice skills. These stations assessed various aspects such as clinical decision-making ability, emergency treatment ability, clinical practice skills, communication and cooperation skills, and team cardiopulmonary resuscitation. The theory exam covered topics including mechanical ventilation, hemodynamics, electrolyte acid-base disorders, and renal replacement therapy.

### Questionnaire survey

The 22 residents in the MR group participated in the questionnaire survey. Participation was anonymous. The survey dealt with the feasibility of the general concept, potential gains in knowledge, effectiveness, encouraging potential, implementation form and assessment of complexity. A 5-point Likert scale (1 = “totally agree”, 3 = “neutral” and 5 = “totally disagree” was chosen. Specific questions of the questionnaire survey are presented in Table 2.

**Table 2:** Survey questions using a 5-point Likert scale.

Question	Description
Question 1	I am interested in participating in the teaching activities of the thematic morning report, as it has sparked my interest in the topics presented.
Questions 2	Compared to traditional teaching ward rounds, the thematic morning report offers more novelty and flexibility.
Questions 3	I have gained new knowledge from the sessions and can apply it in my daily practice.
Questions 4	The discussions and interactions have enriched my knowledge and understanding of the clinical issues.
Questions 5	It is important for the themes to be integrated with clinical practice.
Questions 6	The duration of the thematic morning report session was appropriate.
Questions 7	The timing and the number of participants of the thematic morning report can be adjusted flexibly.

### Statistical analysis

General information of two groups of people was surveyed through Questionnaire Star, and after evaluation, it was sorted and statistically analyzed. Gender, education level, and question classification belong to count data, which are represented by frequency. Age and scores are quantitative data, and after normality testing, they belong to normal distribution data, expressed as mean ± standard deviation. Statistical analysis was conducted using SPSS software version 24.0 (SPSS, Inc., Chicago, Illinois). Chi square test was used to compare categorical variables between groups for categorical data, and one-way ANOVA test was used for quantitative data between two or more groups. To determine whether the learning method of the thematic morning report is an independent factor affecting assessment performance, variables were classified and multiple logistic regression analysis was used to compare the relationship between thematic MR and evaluation results. A *P*-value<0.05 is considered statistically significant.

## Results

### Comparison of general information of the resident physicians

There were no significant differences between the two groups of resident physicians in baseline characteristics, including gender distribution (*P* = 0.373), age (*P* = 0.235), and educational background (*P* = 0.609). Prior to receiving standardized training in critical care medicine, a pre-training test on the theoretical knowledge related to critical care was conducted to understand the basic knowledge mastery of the trainees in the field of critical care medicine. The results indicated that both groups scored similarly in mechanical ventilation, hemodynamics, electrolyte acid-base disorders and team cardiopulmonary resuscitation, with no statistically significant differences observed (*P* > 0.05). This confirms comparable baseline competence between the two groups. The general information of the resident physicians and the results of pre-training assessment are presented in Table 3.

**Table 3:** The general information of the resident physicians and the results of pre-training assessment.

	Thematic MR (n = 22)	Traditional training (n = 15)	*P*
**General information**			
Gender (male/female)	9/13	4/11	0.373
Age (year)	28.27 ± 2.55	27.67 ± 4.68	0.235
Degree (bachelor/graduate)	15/7	9/6	0.609
**Pre-training assessment (score)**			
Mechanical ventilation	65.12 ± 12.53	67.63 ± 18.92	0.359
Hemodynamics	56.31 ± 22.19	50.73 ± 18.66	0.187
Electrolyte and acid base disorders	60.27 ± 17.45	59.27 ± 22.18	0.682
Renal replacement therapy	45.88 ± 10.62	40.79 ± 22.81	0.541
Team cardiopulmonary resuscitation	37.56 ± 8.99	38.27 ± 9.14	0.457

### Evaluation of training effectiveness of the thematic morning report training mode and the traditional training mode

After the standardized training in critical care medicine, the performance was assessed by OSCE stations and theory examinations. The assessment included that clinical decision-making ability, emergency treatment ability, clinical practice skills, communication and cooperation skills and medical theoretical knowledge. The theory exam covered topics including mechanical ventilation, hemodynamics, electrolyte acid-base disorders, and renal replacement therapy. The results showed that the MR group outperformed the traditional training group in terms of clinical decision-making ability (95.21 ± 21.35 vs. 71.43 ± 19.24, *P* = 0.008), emergency treatment ability (97.53 ± 32.65 vs. 77.38 ± 19.58, *P* = 0.013), clinical practice skills (94.21 ± 13.65 vs. 70.35 ± 13.28, *P* = 0.005), and the differences were statistically significant (*P* < 0.05). There was no significant difference between the two groups in terms of communication and teamwork skills (*P* = 0.524) and team cardiopulmonary resuscitation (*P* = 0.256). Theoretical assessment results showed that the MR group scored better than the traditional training group in mechanical ventilation(87.12 ± 12.53 vs. 76.54 ± 22.44, *P* = 0.039), hemodynamics (86.55 ± 25.69 vs. 71.62 ± 18.72, *P* = 0.016), electrolyte acid-base disorders(91.75 ± 18.91 vs. 70.92 ± 34.52, *P* = 0.008), and renal replacement therapy (78.76 ± 9.55 vs. 61.55 ± 18.96, *P* = 0.033). The specific results are shown in Table 4.

**Table 4:** The results of comprehensive assessment after training.

	Thematic MR (n = 22)	Traditional training (n = 15)	*P*
**Practical skills assessment-OSCE (score)**			
Clinical decision-making ability	95.21 ± 21.35	71.43 ± 19.24	0.008
Emergency treatment	97.53 ± 32.65	77.38 ± 19.58	0.013
Clinical practice skills	94.21 ± 13.65	70.35 ± 13.28	0.005
Team cardiopulmonary resuscitation	87.97 ± 18.04	80.31 ± 11.21	0.256
Communication and cooperation skills	76.91 ± 10.33	72.65 ± 13.47	0.524
**Theoretical knowledge assessment (score)**			
Mechanical ventilation	87.12 ± 12.53	76.54 ± 22.44	0.039
Hemodynamics	86.55 ± 25.69	71.62 ± 18.72	0.016
Electrolyte and Acid Base Disorders	91.75 ± 18.91	70.92 ± 34.52	0.008
Renal replacement therapy	78.76 ± 9.55	61.55 ± 18.96	0.033

OSCE, objective structured examination

### Factors influencing the training performance of comprehensive theoretical knowledge

We categorized 37 resident physicians into two groups: the excellent group (scores ≥85 points) and the qualified group (scores ≥60 points and < 85 points) based on comprehensive assessment and training results. The relationship between training model and training results was compared using multiple logistic regression analysis, and the results revealed that utilizing the thematic MR training format is advantageous for enhancing training outcomes (OR 2.518, 95% CI:1.098–3.015, *P* < 0.05, Table 5).

**Table 5:** Multiple regression analysis of the factors influencing the training performance of comprehensive theoretical knowledge.

	Excellent group (n = 17)	Qualified group (n = 20)	Univariate analysis (*P*)	Multivariate analysis [OR (95% CI), *P*]
Gender (male/female)	6/11	7/13	0.985	–
Age (year)	23.76 ± 4.31	30.18 ± 5.37	0.039	1.346 (0.942-1.843), 0.236
Degree (Bachelor/Master)	8/9	16/4	0.047	1.028 (0.934-1.337), 0.467
Training model (Thematic MR/ Traditional)	16/1	4/16	<0.001	2.518 (1.098-3.015), 0.017

### Results of questionnaire survey

A questionnaire (Table 2) in 22 critical care medicine resident physicians was conducted. Compared with the traditional teaching ward rounds, 72.7% (16/22) of residential training physicians are more willing to participate in the teaching form of thematic MR, and they are better than the traditional teaching ward rounds in terms of novelty, flexibility, learning effect of professional knowledge and interaction degree. A significant majority of residential physicians, up to 90.9% (20/22), expressed the necessity of thematic MR in intensive care teaching. Furthermore, 86.4% (19/22) of these physicians believed that the implementation of thematic MR could enhance their proficiency in critical care knowledge. Additionally, 68.2% (15/22) of resident physicians indicated a preference for thematic MR sessions to be conducted from 10:00–11:00 am daily. Moreover, 54.5% (12/22) of the resident physicians perceived that optimal learning outcomes were achieved when the number of participants ranged between 6 and 10 (Table 6). Interestingly, a small percentage, 18.18% (4/22) of participants, suggested that reducing the number of participants to 3–5 could foster more interactive discussions (Table 6).

**Table 6:** The questionnaire survey results.

	Completely agree, n (%)	Agree, n (%)	Neutral, n (%)	Disagree, n (%)	Completely disagree, n (%)
Questions 1	8 (36.36)	8 (36.36)	6 (27.27)	0 (0)	0 (0)
Questions 2	9 (40.91)	7 (31.82)	6 (27.27)	0 (0)	0 (0)
Questions 3	12 (54.55)	8 (36.36)	2 (9.09)	0 (0)	0 (0)
Questions 4	13 (59.09)	6 (27.27)	3 (13.64)	0 (0)	0 (0)
Questions 5	15 (68.18)	5 (22.73)	2 (9.09)	0 (0)	0 (0)
Questions 6	7 (31.82)	8 (36.36)	7 (31.82)	0 (0)	0 (0)
Questions 7	5 (22.73)	7 (31.82)	6 (27.27)	4 (18.18)	0 (0)

## Discussion

Our study demonstrated the feasibility of conducting a thematic MR session centered on critical care medicine in the ICU. Furthermore, our findings suggest that this approach had not only a positive influence on the training outcomes of ICU residents but also a potential to stimulate interest of the trainees in critical care medicine.A recent study [15] also suggests that the implementation of the critical care morning report has significantly improved our residents’ knowledge of guideline-directed management and potential complications, as well as enhanced their confidence in caring for critically ill patients.

Following 24 weeks of specialized training in critical care medicine, residents in the thematic MR group exhibited notable enhancements in their clinical decision-making abilities, practical skills, and understanding of critical care-related theories. Our study revealed that individuals in the the excellent performance group tended to be younger, possibly due to the inclination of young residents to embrace innovation. Additionally, the excellent performance group comprised a higher proportion of residents with master degree compared to bachelor residents, suggesting that the superior self-study and critical thinking skills in residents with master degree may have contributed to their excellent performance. Importantly, multivariate regression confirmed that the thematic MR model independently predicted superior performance (OR = 2.518), reinforcing its value in residency training. Age and degree lost significance in multivariate analysis, implying that training methodology is the primary driver of success. Furthermore, results from the questionnaire survey indicated a strong positive reception towards the thematic MR teaching format. Interestingly, there were no substantial differences between the two groups in terms of non-technical skills such as communication and teamwork. This observation could be attributed to the emphasis placed on theoretical instruction for medical students in both the thematic MR training format and traditional training format, while neglecting the training of non-technical skills. In future, it is essential to enhance training in non-technical skills.

The current MR as implemented in the internal medicine is based on the diagnosis and treatment of diseases, which is related to the clinical diagnosis and treatment mode of internal medicine [16]. Critical care medicine is mainly characterized by a problem-oriented and titrated clinical treatment model. Therefore, we used thematic MR in training practice of critical care medicine. A study indicates that the content of attending teaching comments is likely affected by numerous factors, including local prevalence of disease, attending level of expertise, facilitator choice of topic in scripted reports, and presenter choice of case in unscripted reports [17]. Reassuringly, our results demonstrated that attendings taught broadly across a range of topics. According to the results of the investigation and analysis of the American College of Graduate Medical Education (ACGME) in 2018 [18], we determined that our thematic MR would be implemented with a duration of 30–45 min from 9:00–12:00am from Monday to Friday, and carried out the training activities for the problems encountered in clinical work. In the process of implementation, according to the questionnaire survey of resident physicians who participated in the thematic MR, the time period, frequency, thematic content, categories and number of participants of the thematic MR have been well accepted in the training of intensive resident physicians, and we believe that the thematic MR has a good feasibility and a close combination of theory and practice.The thematic MR may be able to assist in addressing the gaps in the knowledge regarding the management of critical diseases to a certain extent. Meanwhile, it could potentially aid in refining the training objectives and plans based on the conventional training mode, and might offer some guidance for the traditional training. Through the implementation of thematic MR, it was observed that the content of thematic MR exhibits a focused approach, beginning with practical issues and demonstrating a systematic understanding of knowledge. However, it is essential to supplement this with other conventional training activities. Future research should prioritize the investigation of the following key areas [19]. First, it is necessary to describe the mode of learning and training carried out in the MR, to give full play to its characteristics of training and learning, and to complement other teaching activities. Second, the satisfaction of the participants and the motivating factors that play a role in the MR needs to be investigated in order to improve the training quality of the MR. Finally, multi-institutional studies on the effectiveness of conducting new strategies for MR are needed to validate the robustness of interventions beyond project-specific impact.

Our study has several limitations. It is a monocentric study, and results need to be confirmed in a larger population. Nonetheless, while the size of this study was relatively small, it was powerful enough to highlight significant results.The evaluation cycle should be longer.The 24-week training period and immediate post-training assessment may not capture long-term retention of skills or knowledge. Longitudinal follow-ups are necessary to evaluate sustained educational impacts. In the future, addressing these limitations through multi-institutional collaborations, extended follow-up periods, and hybrid training models could strengthen the evidence base and optimize critical care residency training programs.

## Conclusion

Our results align with Ashford Scott et al.[14], who reported that the implementation of the critical care morning report has significantly improved our residents’ knowledge of guideline-directed management and potential complications, as well as enhanced their confidence in caring for critically ill patients. Thematic MR is a unique form of morning report that incorporates elements of critical care medicine training to traditional training. It is easy to implement, flexible, and follows a hierarchical structure in teaching and training critical care resident physicians. The thematic MR training format has been shown to enhance training outcomes and serves as a valuable supplement to conventional training.

Through the continuous development of the theme-based MR training, a complete set of theme-based MR courses and implementation procedures can eventually be formed, so as to achieve standardization and homogenization of intensive care medicine residential training, and further improve the quality of standardized training for critical care medicine residents.

## Supporting information

S1 Data Raw Data. (XLSX)
